# Venous Thromboembolism in Hospitalized Critical and Noncritical COVID-19 Patients: A Systematic Review and Meta-analysis

**DOI:** 10.1055/s-0041-1730967

**Published:** 2021-07-06

**Authors:** Eman M. Mansory, Suthan Srigunapalan, Alejandro Lazo-Langner

**Affiliations:** 1 Division of Hematology, Department of Medicine, Western University, London, Ontario, Canada; 2 Department of Hematology, King Abdulaziz University, Jeddah, Saudi Arabia; 3 Division of Hematology, Department of Medicine, Western University, London, Ontario, Canada; 4 Department of Epidemiology and Biostatistics, Western University, London, Ontario, Canada

**Keywords:** venous thromboembolism, anticoagulation, COVID-19

## Abstract

**Introduction** Venous thromboembolism (VTE) has been observed as a frequent complication in patients with severe novel coronavirus disease 2019 (COVID-19) infection requiring hospital admission.

**Aim** This study was aimed to evaluate the epidemiology of VTE in hospitalized intensive care unit (ICU) and non-ICU patients.

**Materials and Methods** PubMed was searched up to November 13, 2020, and updated in December 12, 2020. We included studies that evaluated the epidemiology of VTE, including pulmonary embolism (PE) and/or deep vein thrombosis (DVT), in patients with COVID-19.

**Results** A total of 91 studies reporting on 35,017 patients with COVID-19 was included. The overall frequency of VTE in all patients, ICU and non-ICU, was 12.8% (95% confidence interval [CI]: 11.103–14.605), 24.1% (95% CI: 20.070–28.280), and 7.7% (95% CI: 5.956–9.700), respectively. PE occurred in 8.5% (95% CI: 6.911–10.208), and proximal DVT occurred in 8.2% (95% CI: 6.675–9.874) of all hospitalized patients. The relative risk for VTE associated with ICU admission was 2.99 (95% CI: 2.301–3.887, *p* <0.001). DVT and PE estimated in studies that adopted some form of systematic screening were higher compared with studies with symptom-triggered screening. Analysis restricted to studies in the 5th quintile of sample size reported significantly lower VTE estimates.

**Conclusion** This study confirmed a high risk of VTE in hospitalized COVID-19 patients, especially those admitted to the ICU. Nevertheless, sensitivity analysis suggests that previously reported frequencies of VTE in COVID-19 might have been overestimated.

## Introduction

The novel coronavirus disease (COVID-19) caused by the severe acute respiratory syndrome coronavirus-2 (SARS-CoV-2) virus was declared as a worldwide pandemic on March 11, 2020 and has so far claimed the lives of more than 2,034,527 people and infected more than 94 million as of January 20, 2021 ( *https://www.who.int/* ). A wide range of presenting symptoms and disease severity has been observed with COVID-19 from asymptomatic to multiorgan failure and death. In patients with severe disease, inflammation is believed to precipitate systematic coagulation derangement that may evolve into overt disseminated intravascular coagulopathy (DIC) and vascular damage. 1 There has been increasing evidence that severe COVID-19 infection increases the risk of venous thromboembolism (VTE), including deep vein thrombosis (DVT) and pulmonary embolism (PE) with important prognostic implications. 2 Although this risk is now well established based on many observational studies, there are uncertainties with regard to the magnitude of the risk and strategies to prevent and manage VTE risk associated with the infection in patients admitted with severe disease. We aimed to systematically review the available evidence on thrombosis risk associated with COVID-19 infection in the intensive care unit (ICU) and non-ICU patients to help guide study design and decision-making in these patients.

## Methods

**Fig. 1:**
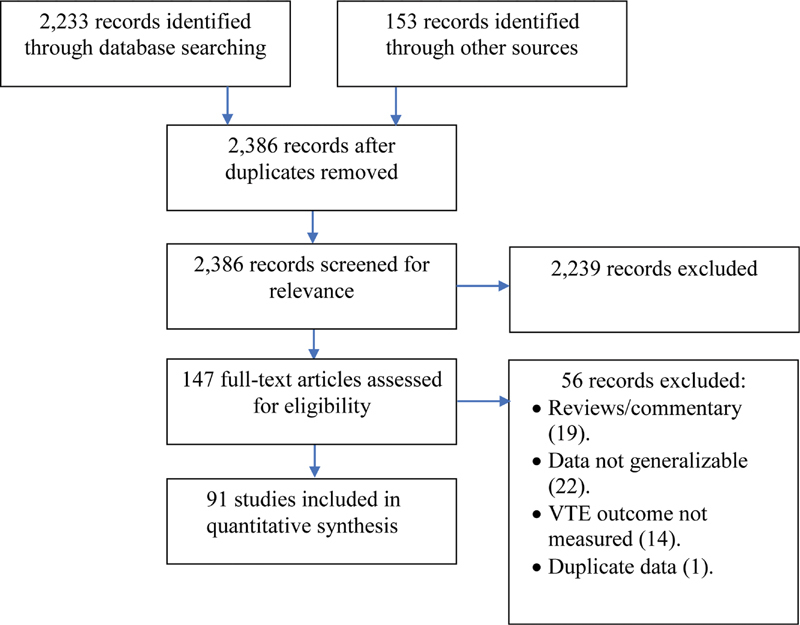
Preferred reporting items for systematic reviews and meta-analyses (PRISMA) flow diagram for study selection. VTE, venous thromboembolism.

This systematic review and meta-analysis was performed following the Preferred Reporting Items for Systematic reviews and Meta-analysis (PRISMA) guidelines ( Fig. 1 ).

The review is registered in Prospero ( *https://www.crd.york.ac.uk/PROSPERO* ), with registration number: CRD42020225318.

We conducted a literature search using a single search engine through PubMed using the Medical Subject Headings (MeSH) “COVID,” “coronavirus,” “coagulopathy,” “disseminated intravascular coagulation,” “hemostasis,” “thrombosis,” “deep vein thrombosis,” “pulmonary embolism,” and “venous thromboembolism” through Boolean operators. We also retrieved additional references from retrieved papers and from the guidelines of the International Society on Thrombosis and Haemostasis (ISTH) 3 and Thrombosis UK ( *https://thrombosisuk.org/covid-19-thrombosis.php* ). Additionally, preprint databases (preprints.org and biorxiv.org) were also searched for papers accepted but not yet published, and we also scanned the retrieved papers for additional references. Related abstracts from the 62nd American Society of Hematology meeting held in December 2020 were included ( *https://ash.confex.com/ash/2020/webprogram/start.html* ).

Full-text articles, letters, brief reports, editorials, abstracts, and correspondence published in 2020 were eligible for inclusion. We included randomized controlled trials (RCTs), observational cohort studies (prospective or retrospective), case-control studies or case series that included adult participants with hospitalized COVID-19 infection (including ICU and non-ICU setting), and assessed VTE incidence/prevalence. We excluded studies that had no original data and studies that included only a specific population of patients that would not reflect the general epidemiology of VTE in COVID-19 patients (e.g., autopsy studies, studies on pregnant patients only, or patients with HIV). There was no language restriction.

Initially, broad screening was conducted according to title. Subsequently, all relevant abstracts were reviewed. In the end, all potentially included articles were reviewed in full length. Two reviewers (E.M.M. and S.S.) separately assessed papers for potential inclusion to verify eligibility. Discrepancies were resolved by consensus and/or in conjunction with a third reviewer (A.L.-L.). Translation of included papers from German to English was conducted with the use of Google Chrome's built-in translation tool.

Data were abstracted on study identifiers, study specific methodological data (including sample size, study design, health care setting, and ultrasound screening strategy), patient- and disease-specific data (hospitalization, ICU admission, disease severity, and thromboprophylaxis) and outcome-specific data including (VTE, DVT, PE, and catheter-related thrombosis).

The primary outcome of this meta-analysis was the proportion of VTE, that is, DVT in upper or lower limps (including catheter-related thrombosis) and PE or the composite of both in ICU and non-ICU patients. The estimate of the primary outcome was reported stratified by health care setting (ICU vs. non-ICU), screening protocol implemented and the thromboprophylaxis strategy used in patients with COVID-19 infections. Of note, as some studies included both proximal and distal DVTs in their results, only proximal events were included in our analysis. Moreover, studies differed in the way events were calculated. Some studies used prevalence while others used incidence rates depending on the study type. In this study, we use the proportion of patients diagnosed with VTE in the included studies (prevalence).

Quality and risk of bias of included observational studies was rated with The Newcastle-Ottawa Scale (NOS) for assessing the quality of nonrandomized studies in meta-analyses. 4 For randomized trials, we used the scale by Jadad et al. 5

### Statistical Analysis

We performed a meta-analysis of proportions for the frequency of VTE to further explore our findings. We estimated pooled proportions through a Freeman–Tukey transformation using fixed and random effect models and generic inverse variance method, as appropriate. Given the statistical heterogeneity, the reported pooled proportions are those obtained by a random effects model. Sensitivity analyses were conducted according to setting (ICU vs. non-ICU), study design, screening strategy, thromboprophylaxis strategy, and sample size quintiles. Heterogenicity between studies was assessed by Cochrane Q and Higgins *I* ^2^ analysis. Publication bias was assessed using Eggers' test and funnel plot. The analysis was done using MedCalc Statistical Software version 19.2.6 (MedCalc Software Ltd., Ostend, Belgium).

## Results

### Search Strategy

The initial search included papers published between January 1, 2020, and November 13, 2020, and was extended on December 12, 2020, to cover for potential studies presented at the 62nd American Society of Hematology meeting. The search yielded 2,233 studies through PubMed and an additional 153 papers from other sources. Following a title and abstract screening, a total of 148 articles were reviewed in full text. Of those, 91 studies fulfilled our eligibility criteria. The 56 excluded studies included 19 literature reviews/systematic reviews/commentary letters, 22 studies that looked at a specific population (thus preventing generalizability), 14 studies that didn't report the incidence of VTE in the study, and 1 study that had duplicate data (a follow-up study). Of the final 91 studies that were included, 70 were cohort (52 retrospective and 25 prospective), 7 were cross-sectional studies, 5 were case series, 1 was a before–after study, and 1 was an RCT. All studies were in English except one study that was in German. 6

A total of 35,017 patients with COVID-19 infection were included, 12,941 were hospitalized non-ICU patients and 8,719 were ICU patients. The rest were hospitalized patients but did not have a clearly identified location. The largest study included 3,334 patients and the smallest had 19 patients.

The majority of the studies (59) came from Europe, 17 from the United States, 5 from China, 4 from South America (Mexico and Brazil), 3 from the Middle East (UAE and Saudi Arabia), 1 from Canada, 1 from Singapore, and 1 international study. Thirty-five studies included ICU patients only, 23 studies had non-ICU patients only, and 33 studies included both ICU and non-ICU patients. Both PE and DVT events were reported in 50 studies, while 19 studies reported on DVT events only and 18 on PE events only. Four studies reported VTE incidence without specifying what type was it. 7 8 9 10 The screening strategy differed between papers: 35 studies used some sort of mandatory screening for VTE (either ultrasound or chest imaging) and 42 studies searched for VTE only if clinically suspected. Thirteen studies did not report their screening strategy and one study crossed patients to mandatory screening after increasing thromboprophylaxis dose. 11

With regard to the thromboprophylaxis strategy used, 40 studies used prophylactic doses of low molecular weight heparin for thromboprophylaxis, 3 studies used an intermediate dose, 14 studies did not specify the regimen used for thromboprophylaxis, and no thromboprophylaxis was used in 3 studies. In 29 studies, the thromboprophylaxis was a combination of different dosing (this was sometimes based on the patients' clinical situation, 12 13 results of Rotational Thromboelastometry (ROTEM), 14 Padua's score, 15 16 D-dimer level, 17 or doses were changed at a certain point of time due to changes in the prophylaxis protocol at the center performing the study 18 ), one study used a prophylactic then an intermediate dose in a before–after study design, 11 and one study randomized patients between therapeutic and prophylactic dosing. 19 Characteristics of included studies are summarized in Table 1 . Additional information on individual studies and a detailed reference list is included in Supplementary Tables S1 and S2 .

**Table 1:** Characteristics of included studies

Characteristics	No of studies
Country	
Europe	49
The United States	17
China	5
South America	4
Middle East	3
Other	3
Study design	
Prospective cohort	25
Retrospective cohort	52
Cross sectional	7
Case series	5
Randomized controlled study	1
Before and after study	1
Setting	
ICU only	35
Non-ICU only	23
Both ICU and non-ICU	33
Events reported	
PE only	18
DVT only	19
Both DVT and PT	50
VTE not specified	4
Screening strategy	
Mandatory screening	35
Symptom triggered	42
Not reported	13
Patient crossed over	1
Thromboprophylaxis strategy	
Prophylactic dosing	40
Intermediate dosing	3
Combined doses	29
No prophylaxis	3
Not reported	14
Patients crossed over	1

Abbreviations: DVT, deep vein thrombosis; ICU, intensive care unit; PE, pulmonary embolism; PT, pulmonary thrombosis; VTE, venous thromboembolism.

There was significant heterogenicity in patients' selection between studies. Although most studies included all patients admitted to hospital with a COVID-19 infection, 13 studies included only patients who had imaging and some studies included only patients with D-dimers above a certain threshold. 7 20 21 Study outcomes varied as well, as some studies included both proximal and distal DVTs, and others both arterial and venous clots. All estimates reported high statistical heterogeneity, and thus we present only the results of random effect models. Funnel plots suggested the presence of reporting bias related with higher standard errors usually seen with smaller sample sizes ( Supplementary Figs. S1–S5 ).

#### Epidemiology of VTE

Of the 35,017 patients from 91 studies included with COVID-19 infection, a total of 2,722 patients had at least one VTE event, with a pooled prevalence estimate of all reported VTE event of 12.8% (95% confidence interval [CI]: 11.103–14.605; Table 2 ). Of these, 1,490 were PE events, 1,074 were DVT (DVT and PE events are not mutually exclusive), and details were not provided in 357. The rate of VTE varies widely between different studies (0–79.41%) likely stemming from the differences in study population, screening protocol, anticoagulation regimen, measured study outcomes, and whether the study included ICU and/or non-ICU patients.

**Table 2:** Overall proportion of COVID-19 patients with venous thromboembolism in different health care settings

Patient population	No. of total patients	Percentage of patients with VTE	95% CI
All patients	35,017	12.827	11.117–14.641
ICU	8,719	24.055	20.070–28.280
Non-ICU	12,941	7.724	5.956–9.700

Abbreviations: CI, confidence interval; COVID-19, novel coronavirus disease 2019; ICU, intensive care unit; VTE, venous thromboembolism.

The prevalence of VTE decreased as the study population size increased. Compared with the overall estimate, the prevalence of VTE for studies with a sample size in the 5th quintile was 5.5% (95% CI: 4.281–6.850). Results of other sensitivity analysis are shown in Table 3 .

**Table 3:** Proportion of COVID-19 patients with venous thromboembolism: sensitivity analysis of all hospitalized patients

Subcategories in all hospitalized patients	No. of total Patients	Percentage of patients with VTE	95% CI
Study type			
• Prospective	4,661	11.888	7.444–17.203
• Retrospective	28,006	11.470	9.737–13.325
• Cross sectional	838	13.597	5.419–24.721
• Case series	1,420	34.015	7.494–67.865
• Other design	92	29.870	21.133–39.417
Screening mode			
• Mandatory	6,141	18.587	14.018–23.638
• Symptom triggered	18,623	11.522	9.224–14.039
Thromboprophylaxis			
• No prophylaxis	1,289	13.449	5.209–24.750
• Prophylactic dose	15,220	12.899	10.344–15.691
• Intermediate dosing	186	10.450	0.545–30.445
• Multiple dosing	14,550	12.705	10.003–15.678
• Prophylaxis not reported	3,762	12.838	7.578–19.234
Sample size			
• 5th quintile	27,569	5.495	4.281–6.850

Abbreviations: CI, confidence interval; COVID-19, novel coronavirus disease 2019; VTE, venous thromboembolism.

#### Intensive Care Unit versus non-Intensive Care Unit

In the analysis restricted to ICU patients, the overall proportion of ICU patients who had a VTE is 24.1% (95% CI: 20.070–28.280). For non-ICU patients, the overall proportion of patients who had a VTE is 7.7% (95% CI: 5.956–9.700). ICU patients had a relative risk of VTE of 2.99 compared with non-ICU patients (95% CI: 2.301–3.887, *p* < 0.001). Sensitivity analyses according to study type, thromboprophylaxis strategy, screening method, and sample size can be found in Tables 4 and 5 .

**Table 4:** Proportion of ICU COVID-19 patients with venous thromboembolism: sensitivity analysis

Subcategories in ICU patients	No. of total patients	Percentage of patients with VTE	95% CI
Study type			
• Prospective	881	28.566	20.034–37.950
• Retrospective	7,363	20.454	16.135–25.142
• Cross sectional	184	19.217	11.586–28.237
• Case series	199	45.284	22.200–69.501
• Unclear study design	92	29.870	21.133–39.417
Screening mode			
• Mandatory	1,135	33.612	24.504–43.381
• Symptom triggered	4,029	20.618	16.162–25.463
Thromboprophylaxis			
• Prophylactic dose	3,536	22.522	17.909–27.498
• Intermediate dosing	81	19.245	11.538–28.370
• Multiple dosing	4,462	26.709	17.692–36.829
• Prophylaxis not reported	549	24.402	19.786–29.336
Sample size			
• 5th quintile	5,874	15.708	10.668–21.515

Abbreviations: CI, confidence interval; COVID-19, novel coronavirus disease 2019; ICU, intensive care unit; VTE, venous thromboembolism.

**Table 5:** Proportion of non-ICU COVID-19 patients with venous thromboembolism: sensitivity analysis

Subcategories in non-ICU patients	No. of total patients	Percentage of patients with VTE	95% CI
Study type			
• Prospective	1,511	8.795	4.294–14.699
• Retrospective	11,007	6.485	4.572–8.705
• Cross sectional	409	11.695	5.602–19.636
Screening mode			
• Mandatory	3,170	11.152	7.437–15.509
• Symptom triggered	9,771	5.504	3.971–7.270
Thromboprophylaxis			
• No prophylaxis	1,208	10.453	2.340–23.399
• Prophylactic dose	9,295	6.899	5.000–9.078
• Multiple dosing	1,840	7.956	4.577–12.166
• Prophylaxis not reported	493	11.027	5.296–18.513
Sample size			
• 5th quintile	9,988	5.647	3.511–8.252

Abbreviations: CI, confidence interval; COVID-19, novel coronavirus disease 2019; ICU, intensive care unit; VTE, venous thromboembolism.

#### Pulmonary Embolism and Deep Vein Thrombosis

The overall prevalence of PE in all hospitalized patients was 8.5% (95% CI: 6.911–10.208), while DVT was found in 8.2% (95% CI: 6.675–9.874). Unfortunately, we could not extract exact information in ICU and non-ICU patients as many studies reported rates of PE without clearly separating the results according to patient setting.

The rate of DVT in studies that used systematic screening was more than double than the rates observed in studies in which ultrasound was only done when triggered by symptoms (13.5% [95% CI: 8.821–19.572] vs. 6.2% [95% CI: 4.485–8.081]). The same was observed for PE when looking at rates of PE in studies where all patients had to get a computed tomography pulmonary angiogram (CTPA) versus studies where CTPA was only done when patients had respiratory decompensation suggestive of a PE (14.3% [95% CI: 10.091–19.144] vs. 7.5% [95% CI: 5.632–9.563]).

#### Sensitivity Analyses

The sensitivity analyses suggested that VTE estimates in studies are influenced by study design, screening strategy, and particularly by study sample size. Pooled estimates of VTE in all patients, ICU, and non-ICU patients are significantly lower when restricted to studies in the 5th quintile ( Tables 3 4 5 ). The use of different thromboprophylaxis strategies did not show a clear difference in the reported VTE estimates.

## Discussion

With the emergence of COVID-19, a signal for increased risk of thromboembolism was observed in multiple cohort studies. Here, we systematically searched and analyzed the pooled prevalence of VTE in studies that looked at hospitalized COVID-19 patients. Our meta-analysis demonstrates that the VTE risk is a significant concern in critically ill patients (risk ratio [RR] = 2.99, 95% CI: 2.301–3.887); however, non-ICU hospitalized patients still had a significant risk.

The overall estimates of VTE reported in our study are similar to those reported in other reviews. A large systematic review that included 66 papers reported a VTE prevalence of 7.9% (95% CI: 5.1–11.2) in non-ICU and 22.7% (95% CI: 18.1–27.6) in ICU patients, 22 and a second study including 36 studies found the VTE prevalence in non-ICU patients was 10% (95% CI: 6–14%) and in ICU patients 28% (95% CI: 22–34%). 23 In our study, the overall proportions of VTE in all patients, ICU, and non-ICU patients were 12.8, 24, and 7.7%, respectively. However, an analysis of the funnel plots of the estimates consistently showed the possibility of reporting bias associated with higher standard errors. Therefore, a sensitivity analysis including only studies in the 5th quintile for sample size was conducted and showed that the VTE estimates in these studies, although still elevated, were significantly lower at 5.5, 15.7, and 5.6%, respectively. These results suggest that it is very possible that the frequency of VTE in COVID-19 patients might have been overestimated in other studies, which could potentially impact the design and interpretation of studies assessing therapeutic interventions in particular anticoagulants.

Our analysis also showed that studies using mandatory screening for VTE (weather by chest CT scans or Doppler ultrasonography) or studies in which only patients who had imaging were included had higher rates of VTE than studies in which screening was triggered by symptoms/treating physician judgment ( Tables 3 4 5 ). Expert guidelines 3 24 suggest against routine screening for DVT and instead to maintain a low threshold for performing ultrasound in patients with a reasonable degree of clinical suspicion for VTE. We also noted that in some of the studies in which a very high rate of DVT was reported, this was attributed to the fact that in some of them distal DVT was included in the total number of VTE events. 25 26 27 28 In our study, we included only proximal DVT events since distal DVT has different connotations when it comes to clinical relevance and need for treatment. 29 On the other hand, the study includes subsegmental PEs given that it was not possible to separate them from segmental episodes in many studies, and it was unclear if the subsegmental events were multiple, symptomatic, or associated with DVT. Furthermore, it is possible that the presence of subsegmental events might have a completely different relevance in patients with COVID-19 given the emerging evidence, suggesting that COVID-19 causes pulmonary intravascular coagulopathy leading to in situ pulmonary thrombosis rather than embolism. 30

Although previous coronavirus epidemics caused by the severe acute respiratory syndrome coronavirus-1 (SARS-CoV-1) and the Middle East Respiratory Syndrome coronavirus (MERS-CoV) were also reported to induce a coagulopathy and thrombotic complications, VTE occurrence associated with COVID-19 seems to be higher. 31 Like many other infectious processes in critically ill patients, the increased risk of VTE in COVID-19 patients is secondary to the activation of the host defense system, leading to activation of coagulation and thrombin generation in addition to suppressed fibrinolysis. This in addition to the severe inflammation, immobilization and endotheliopathy, as well as other patient-specific risk factors form a suitable environment that can lead to VTE. 1 However, the absolute risk of VTE in COVID-19 and how it compares to other inflammatory illnesses remains unclear. Previous studies on patients with severe sepsis or septic shock (non-COVID-19 related) report a high frequency of VTE at 37.2% despite the use of guideline recommended thromboprophylaxis. 32 In addition, it is known that general ICU patients frequently fail VTE prophylaxis (4.45, 7.14, and 7.53% at 7, 14, and 21 days, respectively). 33 On the other hand, some studies that compared the VTE incidence among COVID-19 patients in ICU to ICU patients with other conditions have found a higher incidence of VTE in COVID-19 patients, 6 34 35 while others did not find this. 36 37 38

There is also concern regarding the risk of VTE among COVID-19 patients after discharge from hospital, but current information does not support this. A study showed that 2.6% of discharged patients who do not have an indication for anticoagulation developed a VTE 42 days after discharge. 39 Another showed a 2.5% risk at 30-day postdischarge, 40 and a third study compared the rate of postdischarge VTE in COVID-19 patients (4.8 per 1,000 discharges) with rates of VTE in medical patients postdischarge in 2019 (3.1 per 1,000 discharges), with an odds ratio for postdischarge VTE in COVID-19 of 1.6 (95% CI: 0.77–3.1) indicating that COVID-19 hospitalization does not appear to increase the risk of postdischarge VTE compared with hospitalization from other acute medical illness. 41 More studies are needed to establish the true risk in this population and the appropriate approach needed to mitigate this risk, if any.

In addition to the sepsis-induced hypercoagulability as a cause of increased VTE in COVID-19 patients, many reports suggest the possibility of in situ pulmonary thrombosis rather than PE secondary to the viral pneumonia itself causing local inflammation and pulmonary vasculopathy. This was initially described by McGonagle et al. 30 In this theory, SARS-CoV-2 binds to angiotensin-converting enzyme 2 (ACE2) receptors on type-II pneumocytes and possibly on vascular endothelial cells and causes lysis of the cells immediately leading to direct activation of the endothelium causing procoagulant activity and activates accumulation of fibrin deposits in pulmonary microcapillary venous vessels. 30 42 This was termed pulmonary intravascular coagulopathy (PIC) which is an immune system–mediated thrombosis and distinct from classical DIC. This was supported by autopsy studies that found diffuse alveolar damage and extensive fibrin thrombi in distended small vessels and capillaries, 43 as well as clinical studies that demonstrated chiefly segmental or subsegmental events without concomitant proximal DVT of the lower limbs. 44 45 46 The results of our systematic review suggest an unusually high frequency of PE, compared with that of DVT which is usually two- to three-fold higher in other settings. 47 Interestingly, this tendency of inducing a coagulopathy in patients with COVID-19 was not observed in the pediatric population even in the most severely affected patients and in those with multisystem inflammatory syndrome in children (MIS-C), 48 49 50 and so guidelines only suggest prophylactic anticoagulation in pediatric patients with superimposed risk factors or those with significantly elevated D-dimer (≥5 times the upper limit of normal values). 51

Debates are still ongoing with regard to what protocol of anticoagulation is the most appropriate in adults given the increased risk of VTE in hospitalized COVID-19 patients. The CHEST guidelines 24 and the ISTH guidelines 3 both suggest the use of standard dose anticoagulant thromboprophylaxis over intermediate- or full-dose anticoagulation. Although most of the current focus is on the VTE risk, consideration should be given to the bleeding risk associated with higher doses of anticoagulation in hospitalized and critically ill patients and finding the balance between those two concerns is of the utmost importance. Moreover, with immune thrombosis as a mechanism for the high frequency of VTE in COVID-19 patients, and since we don't use higher doses of anticoagulation in other forms of microangiopathy, increasing the dose of anticoagulation would not be of great effectiveness, 52 and multiple studies have shown increased risk of VTE even in the population that did receive a therapeutic dose of anticoagulation. 35 53 The studies included in our review used many different thromboprophylaxis regimens and many studies used different schedules at different times, and thus it is not possible to obtain any conclusions in this regard. Currently, a large number of randomized controlled trails are ongoing to answer questions on the incidence and prevalence of VTE in COVID-19 patients and the effect of different doses of anticoagulants on VTE risk and overall mortality and morbidity (e.g., NCT04362085, NCT04345848, NCT04359277, NCT04344756, NCT04360824, NCT04359212, NCT04486508, and NCT04512079) and many more. So far, it has been recently reported that three large RCT studies looking at the benefits of full-dose anticoagulation in moderately and critically ill COVID-19 patients (REMAP-CAP, ATTACC, and ACTIV-4) have paused enrollment of critically ill ICU patients due to a concern for futility, as patients on full- dose anticoagulation seem to had a higher rate of bleeding and a potential for harm was observed in this subgroup. 54

## Limitations

The limitations of our study are mainly derived from the heterogeneity of the included studies regarding clinical setting, sample size, population, VTE prophylaxis protocol, and the screening strategy. Given these limitations we were unable to perform an analysis of incidence rather than prevalence. Most importantly, no uniform methodology was used and operational definitions for predictors, outcomes, and follow-up are widely different. To overcome this obstacle, a collaboration between the American Society of Hematology and the ISTH has recently proposed a toolkit of data elements with the aim to support and enhance the process of data collection of thrombosis events in COVID-19 clinical studies. 55

## Conclusion

In conclusion, this is the most comprehensive systematic analysis to date that has aimed to identify the true prevalence of VTE in patients with COVID-19 who are admitted to the hospital. Our findings suggest that the overall VTE estimates albeit high, may be overestimated and further studies using standard definitions and methodology are needed.

## Highlights

- Overall VTE frequency in hospitalized novel coronavirus disease 2019 (COVID-19) patients was 12.8%.
- Venous thromboembolism (VTE) frequency in intensive care unit (ICU) patients was 24.1%.
- In non-ICU hospitalized patients, 7.7% developed VTE.
- Sensitivity analyses suggested that VTE frequency might be overestimated.
