# Supplementary material for: Venous Thromboembolism in Hospitalized Critical and Noncritical COVID-19 Patients: A Systematic Review and Meta-analysis
Source: TH Open. 2021 Jul 6;5(3):e286–94. doi: 10.1055/s-0041-1730967 (PMC8260281; doi:10.1055/s-0041-1730967)
Supplement: Supplementary file 1 — Supplementary Material [file 10-1055-s-0041-1730967-s210023.pdf]

Search Strategy

1-Covid or (coronavirus)
2-Coagulopathy OR (disseminated intravascular coagulation) OR hemostasis
3-Thrombosis OR (deep vein thrombosis) OR (pulmonary embolism) OR (venous thromboembolism)
4-#1 AND (#2 OR #3)
5-1/1/2020 to 13/11/2020
6-#4 AND #5

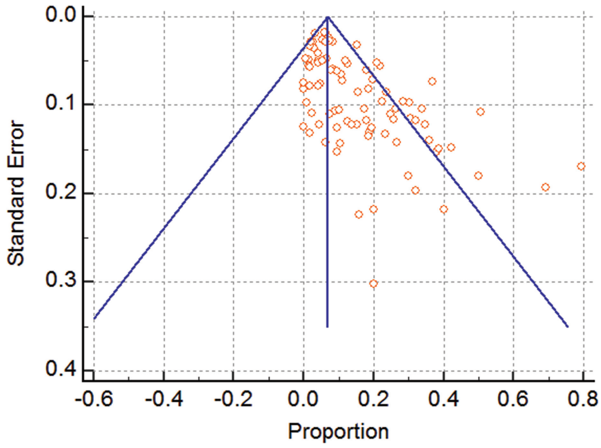

Supplementary Figure S1 Funnel Plot for all patients

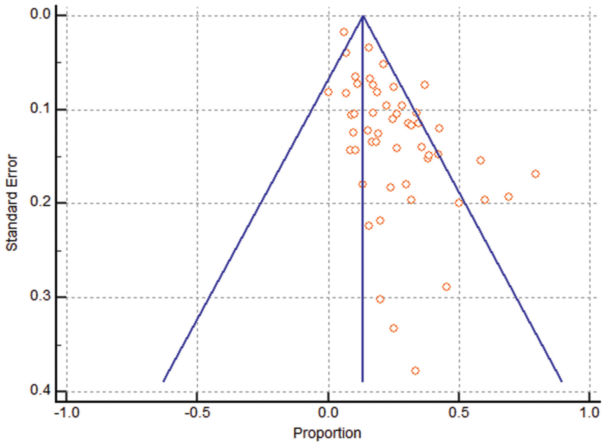

Supplementary Figure S2 Funnel Plot for ICU patients

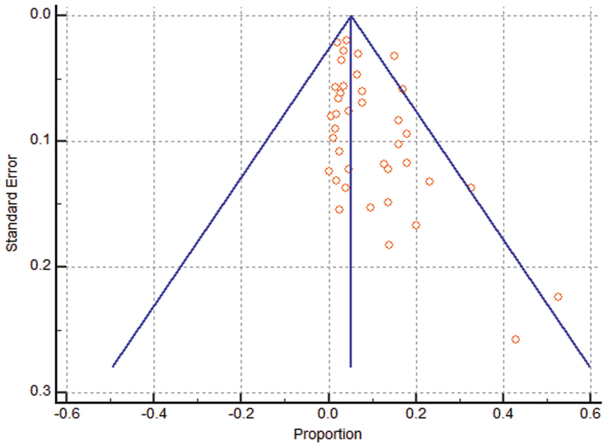

Supplementary Figure S3 Funnel Plot for non-ICU patients

 © 2021. The Author(s).

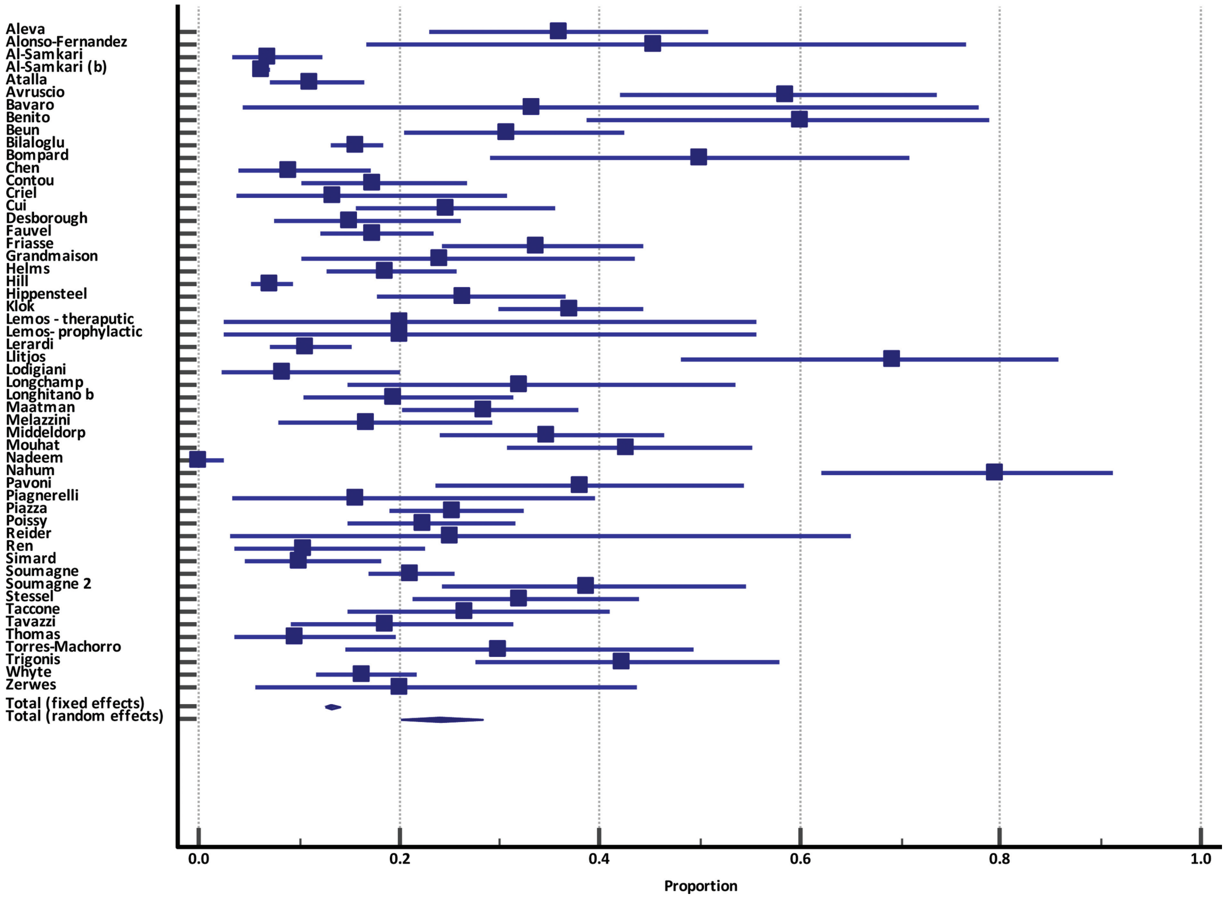

Supplementary Figure S4 Forest Plot showing proption of ICU patients with VTE. **I^2 value of 93.80%, CI (92.60 to 94.81)**

 © 2021. The Author(s).

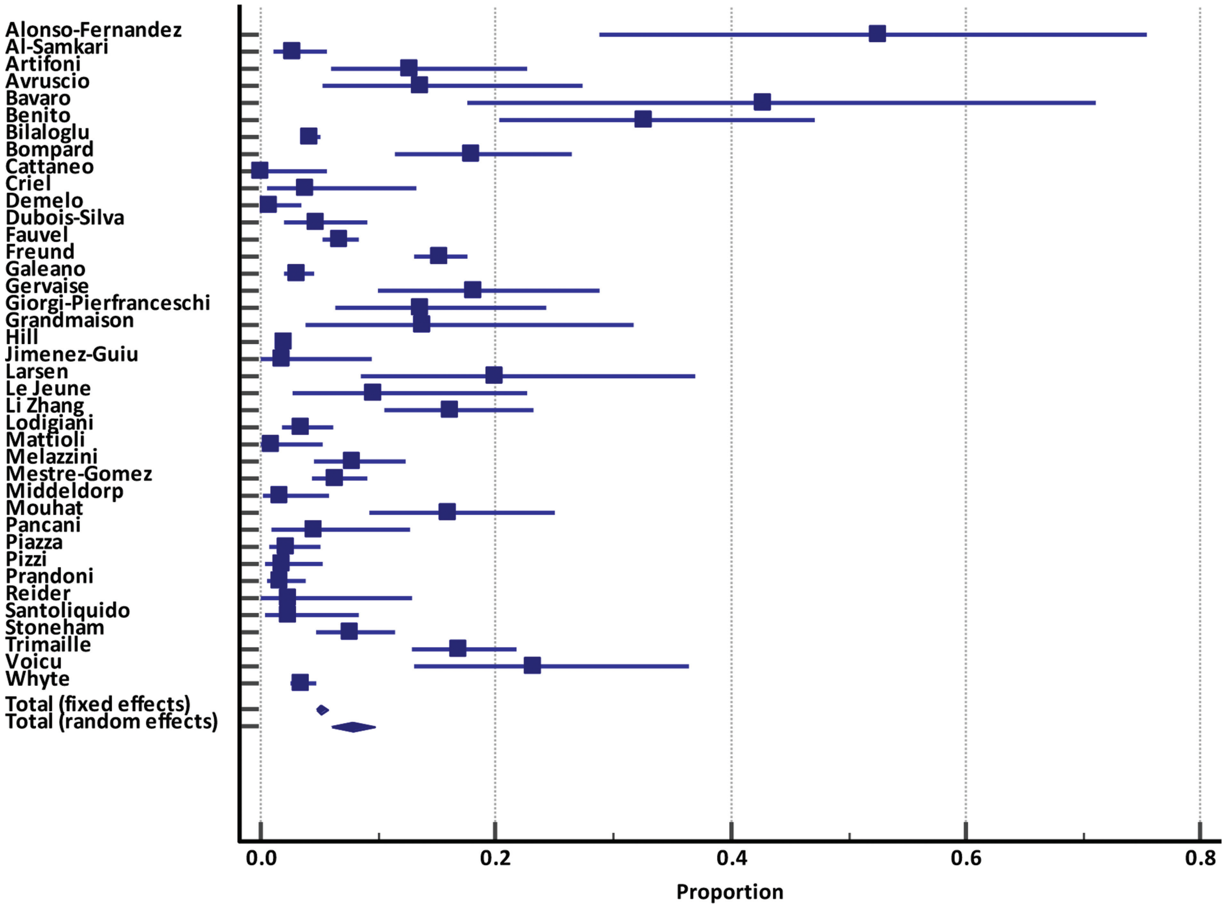

Supplementary Figure S5 Forest Plot showing propotion of non-ICU patients with VTE. **I^2 value of 92.46%, CI (90.61 to 93.95)**

 © 2021. The Author(s).

Supplementary Table S1 Study design and setting

Author	Country	Sample Size	Study design	Health care settings	VTE screening	Prophylaxis regimen	VTE (%)	PE (%)	DVT (%)
Al-Samkari[1]	USA	400	Retrospective cohort study	Hospitalized	No	90% on prophylactic anticoagulation	4.3	2.5	2.5
Artifoni[2]	France	71	Retrospective cohort study	Non-ICU, with ultrasound	Yes	99% on prophylactic anticoagulation	12.7	9.9	2.8
Beun[3]	Netherlands	75	Prospective Cohort study	ICU	No	NR	30.7	26.7	4
Bilaloglu[4]	USA	3334	Retrospective cohort study	Hospitalized	No	Most patients on prophylactic anticoagulation	6.1	3.2	3.9
Bompard[5]	France	135	Retrospective cohort study	Hospitalized, with CTPA	No	Prophylactic dose in non-ICU patients and intermediate dose in ICU patients	23.7	23.7	0
Chen[6]	China	88	Retrospective cohort study	ICU, with U/S	yes	Prophylactic anticoagulation	9.1	0	9.1
Cui[7]	China	81	Retrospective cohort study	ICU	No	No anticoagulation	24.7	0	24.7
Demelo-Rodríguez[8]	Spain	156	Prospective cohort study	Non-ICU, D-Dimer >1000	Yes	100% on prophylactic anticoagulation	0.6	0	0.6
Desborough[9]	UK	66	Retrospective cohort study	ICU	No	100% on prophylactic anticoagulation	15.2	7.6	9.1
Dubois-Silva[10]	Spain	171	Cross-sectional observation study	Non-ICU	No	100% on prophylactic anticoagulation	4.7	4.7	0
Fauvel[11]	France	1240	Retrospective cohort study	Hospitalized, with CTPA	Yes	63% on prophylactic dose and 8.4% on intermediate dose	8.3	8.3	1.5
Freund[12]	6 countries	974	Retrospective cohort study	ER patients who had CTPA	Yes	No anticoagulation	15.2	15.2	0
Fraissé[13]	France	92	Retrospective cohort study	ICU	No	47% on prophylactic dose and 53% on full dose	33.7	27.2	13
Gervaise[14]	France	72	Retrospective cohort study	ER, with CTPA	Yes	No anticoagulation	18.0	18.1	0
Helms[15]	France	150	Prospective cohort study	ICU	No	Prophylactic dose	18.7	16.7	2
Hippensteel[16]	USA	91	Retrospective cohort study	ICU	NR	NR	26.4	5.5	20.9
Klok[17]	Netherlands	184	Prospective cohort study	ICU	No	Prophylactic dose	36.9	35.3	1.6
Koleilat[18]	USA	135	Retrospective case control study	Hospitalized	No	Multiple doses administered	15.6	2.2	13.3
Larsen[19]	France	35	Prospective cohort study	Hospitalized returned travelers	yes	NR	4.2	3.0	1.2
Llitjos[20]	Italy	26	Retrospective cohort study	ICU	Yes	31% had prophylactic anticoagulation and 69% had full dose	69.2	23.1	69.2
Lodigiani[21]	Italy	362	Retrospective cohort study	Hospitalized	No	Multiple doses administered	4.1	2.8	1.4
Longchamp[22]	Switzerland	25	Retrospective cohort study	ICU	Yes	Prophylactic anticoagulation	32	20	24
Mestre-Gomez[23]	Spain	452	Retrospective cohort study	Non-ICU	No	Prophylactic anticoagulation	6.4	6.4	0
Monfardini[24]	Italy	1207	Case series	Hospitalized	No	Prophylactic anticoagulation	2.2	2.2	0.3

(*Continued*)

 © 2021. The Author(s).

Supplementary Table S1 (*Continued*)

Author	Country	Sample Size	Study design	Health care settings	VTE screening	Prophylaxis regimen	VTE (%)	PE (%)	DVT (%)
Nahum[25]	France	34	Case series	ICU	Yes	Prophylactic anticoagulation	79.4	0	79.4
Patell[26]	USA	398	Retrospective cohort study	Hospitalized	NR	Multiple doses administered	5.3	1.8	3.5
Poissy[27]	France	107	Cross sectional	ICU	No	Prophylactic anticoagulation	22.4	20.7	4.7
Poyiadji[28]	USA	328	Retrospective cohort study	328 Hospitalized, with CTPA	Yes	NR	21.9	21.9	0
Rieder[29]	Germany	49	Prospective Cohort study	Hospitalized	No	NR	6.1	4.1	2.0
Ren[30]	China	48	Cross-sectional study	ICU	Yes	Prophylactic anticoagulation	10.4	0	10.4
Santoliquido[31]	Italy	84	Prospective Cohort study	Non-ICU	yes	Prophylactic anticoagulation	2.4	0	2.4
Soumagne[32]	France and Belgium	375	post hoc analysis of COVADIS	ICU	No	NR	21.1	14.7	9.3
Stoneham[33]	UK	274	Retrospective cohort study	Non-ICU	No	NR	7.7	5.8	1.8
Trigonis[34]	USA	45	Retrospective cohort study	ICU, with U/S	Yes	Prophylactic anticoagulation	42.2	0	42.2
Trimaille[35]	France	273	Retrospective cohort study	Non-ICU	No	Prophylactic anticoagulation	17.9	15.4	4.4
Voicu[36]	France	56	Prospective cohort study	ICU	Yes	Prophylactic anticoagulation	23.2	0	23.2
Whyte[37]	UK	1477	Retrospective cohort study	Hospitalized	No	Prophylactic anticoagulation	5.4	5.4	0
Zerwes[38]	Germany	20	Prospective cohort study	ICU	Yes	Prophylactic anticoagulation	20	0	20
Li Zhang[39]	China	66	Cross sectional study	Non-ICU, with U/S	No	NR	34.8	0	34.8
Maatman[40]	USA	109	Case series	ICU	No	Prophylactic anticoagulation	28.4	4.6	27.5
Thomas[41]	UK	63	Retrospective cohort study	ICU	No	Prophylactic anticoagulation	9.5	7.9	1.6
Grillet[42]	France	280	Retrospective cohort study	Hospitalized	No	NR	8.2	8.2	0
Alharthy[43]	Saudi Arabia	89	Prospective cohort study	ICU	Yes	NR	0	24.7	16.9
Hanif[44]	USA	921	Retrospective cohort study	Hospitalized	No	Multiple doses administered	1.7	0	0
Le Jeune[45]	France	42	Retrospective cohort study	Non-ICU	Yes	Multiple doses administered (60% on prophylactic dose, 23% on intermediate and 17% on full dose)	9.5	9.5	2.4
Leonard-lorant[46]	France	106	Retrospective cohort study	Hospitalized, with CTPA	Yes	NR	30.2	30.2	0
Aleva[47]	Netherlands	50	Case series	ICU	No	Prophylactic anticoagulation	36	26	14
Ierardi[48]	Italy	234	Prospective cohort study	Hospitalized	Yes	Prophylactic anticoagulation	10.7	0	10.7
Bavaro[49]	Italy	20	Case series	Hospitalized, with CTPA	Yes	Prophylactic anticoagulation	40	40	0
Criel[50]	Belgium	82	Prospective cohort study	Hospitalized	Yes	Prophylactic anticoagulation	7.3	0	7.3
Mattioli[51]	Italy	105	Retrospective cohort study	Non-ICU	No	Intermediate dose anticoagulation	0.95	0.9	0
Middledrop[52]	Netherlands	198	Retrospective cohort study	Hospitalized	No	Non-ICU on prophylactic dose and ICU patients on intermediate dose	19.7	6.6	13.1
Piagnerelli[53]	Belgium	19	Prospective cohort study	ICU	NR	Intermediate dose anticoagulation	15.8	0	0

Supplementary Table S1 (*Continued*)

Author	Country	Sample Size	Study design	Health care settings	VTE screening	Prophylaxis regimen	VTE (%)	PE (%)	DVT (%)
Stessel[54]	Belgium	72	Before and after study	ICU	Yes	Before: prophylactic dose, After: full dose anticoagulation	31.9	0	31.9
Taccone[55]	Belgium	49	Retrospective analysis of prospective data	ICU	No	Multiple doses administered (45% on prophylactic dose and 37% on intermediate dose)	26.5	26.5	0
Tavazzi[56]	Italy	54	Prospective cohort study	ICU	NR	Prophylactic anticoagulation	18.5	3.7	14.8
Bi[57]	China	420	Prospective cohort study	Hospitalized	NR	NR	1.4	0	1.4
Cattaneo[58]	Italy	64	Prospective cohort study	Non-ICU	Yes	Prophylactic anticoagulation	0	0	0
Galeano-Valle[59]	Spain	785	Prospective cohort study	Non-ICU	No	Prophylactic anticoagulation	3.1	1.9	1.7
Grandmaison[60]	Switzerland	58	Cross sectional study	Hospitalized	Yes	Prophylactic anticoagulation	18.9	6.9	12.1
Giorgi-Pierfranceschi[61]	Italy	66	Cross sectional study	Non-ICU with U/S	Yes	Prophylactic anticoagulation	13.6	0	13.6
Contou[62]	France	92	Retrospective cohort study	ICU	No	Prophylactic anticoagulation	17.4	17.4	0
Alonso-Fernández[63]	Spain	30	Prospective cohort study	Hospitalized, with high D-Dimer and CTPA	Yes	Prophylactic anticoagulation	50	50	0
Benito[64]	Spain	1275	Prospective cohort study	Hospitalized	Yes	Prophylactic anticoagulation	2.5	2.5	0
Atalla[65]	UAE	188	Retrospective cohort study	ICU, U/S if D-Dimer high	Yes	Multiple doses administered	11.2	4.8	6.4
Avruscio[66]	Italy	85	Prospective cohort study	Hospitalized	Yes	Multiple doses administered based on ROTEM	50.6	4.7	30.6
CHOI[67]	USA	1739	Retrospective cohort study	Hospitalized	No	Multiple doses administer (changed after April to intermediate dose)	7.1	2.4	5.5
Hill[68]	USA	2748	Retrospective cohort study	Hospitalized	No	Prophylactic anticoagulation in patient with high Padua score	3.1	1.3	1.5
Jimenez-Guiu[69]	Spain	57	Prospective cohort study	Non-ICU	Yes	Multiple doses administered	1.8	0	1.8
Lemos[70]	Brazil	20	Randomized controlled trail	ICU	No	One prophylactic arm and one full dose arm	20	5	15
Longhitano[71]	Italy	62	Retrospective cohort study	ICU	NR	Intermediate dose anticoagulation	19.4	17.7	1.6
Longhitano[72]	Italy	74	Prospective cohort study	Non-ICU	Yes	Multiple doses administered	25.7	12.2	13.5
Melazzini[73]	Italy	259	Retrospective cohort study	Hospitalized	No	92% on prophylactic anticoagulation	9.7	1.5	8.1
Mouhat[74]	France	349	Retrospective cohort study	Hospitalized	No	Multiple doses administered	12.6	12.6	0
Nadeem[75]	UAE	149	Retrospective cohort study	ICU	No	Multiple doses administered in the same patient	0	0	0
Pancani[76]	Italy	66	Prospective cohort study	Non-ICU	Yes	Prophylactic anticoagulation	4.5	1.5	3.0
Pavoni[77]	Italy	42	Retrospective cohort study	ICU	Yes	Multiple doses administered (intermediate dose if D-Dimer < 3000 and full dose if D-Dimer > 3000)	38.1	7.1	30.9

(*Continued*)

 © 2021. The Author(s).

Supplementary Table S1 (*Continued*)

Author	Country	Sample Size	Study design	Health care settings	VTE screening	Prophylaxis regimen	VTE (%)	PE (%)	DVT (%)
Piazza[78]	USA	399	Retrospective cohort study	Hospitalized	No	Prophylactic anticoagulation	12.0	2.0	10.0
Pizzi[79]	Italy	162	Retrospective cohort study	Non-ICU	Yes	No anticoagulation, screening done on admission	1.9	0	1.9
Prandoni[80]	Italy	308	Retrospective cohort study	Non-ICU	No	Prophylactic anticoagulation	1.6	0.6	0.9
Soumagne 2[81]	France	44	Retrospective cohort study	ICU, with CTPA	Yes	Multiple doses administered	38.6	38.6	0
Torres-Machorro[82]	Mexico	30	Prospective cohort study	ICU	Yes	Multiple doses administered based on Padua score (intermediate and prophylactic)	30	0	30
Al-samkari 2[83]	USA	3239	Retrospective cohort study	ICU with D-Dimer	NR	Multiple doses administered	6.3	0	0
berg[84]	USA	2180	Retrospective cohort study	Hospitalized	NR	Multiple doses administered	6.1	0	0
Marquez[85]	Mexico	447	Prospective cohort study	Hospitalized	No	Multiple doses administered	0.7	0.4	0.2
Tan[86]	Singapore	181	Retrospective cohort study	Hospitalized	NR	NR	0	0	0
Warrior[87]	USA	1265	Prospective cohort study	Hospitalized	NR	Multiple doses administered	6.2	4.8	5.6
Theprungsirikul[88]	USA	1352	Retrospective cohort study	Hospitalized	NR	NR	7.5	3.3	5.1
Camille[89]	Canada	90	Retrospective cohort study	ICU	NR	Multiple doses administered	10	10	3.3
Kodama[90]	USA	580	Retrospective cohort study	Hospitalized	NR	Multiple doses administered	4.1	0.7	3.4
Jiménez-Soto[91]	Mexico	322	Cross sectional study	hospitalized	NR	Multiple doses administered	1.6	1.6	0

Abbreviations: CTPA, computed tomography pulmonary angiogram; DVT = Deep venous thrombosis; ICU, intensive care unit; NR, Not reported; PE, pulmonary embolism; U/S, ultrasonography; UAE, United Arab Emirates; USA, United States of America; VTE, venous thromboembolism.

 © 2021. The Author(s).

Supplementary Table S2 Quality of identified studies

Author	Study design	Health care settings	Selection	Compatibility	Outcome
Al-Samkari[1]	Retrospective cohort study	Hospitalized	****	*	**
Artifoni[2]	Retrospective cohort study	Non-ICU, with ultrasound	***	**	*
Beun[3]	Prospective Cohort study	ICU	****	*	*
Bilaloglu[4]	Retrospective cohort study	Hospitalized	****	**	**
Bompard[5]	Retrospective cohort study	Hospitalized, with CTPA	***	*	**
Chen[6]	Retrospective cohort study	ICU	***	**	*
Cui[7]	Retrospective cohort study	ICU	****	**	**
Demelo-Rodríguez[8]	Prospective cohort study	Non-ICU, D-Dimer >1000	***	*	**
Desborough[9]	Retrospective cohort study	ICU	****	**	**
Dubois-Silva[10]	Cross-sectional observation study	Non-ICU	****	**	*
Fauvel[11]	Retrospective cohort study	Hospitalized, with CTPA	***	*	**
Freund[12]	Retrospective cohort study	ER patients who had CTPA	**	*	**
Fraissé[13]	Retrospective cohort study	ICU	****	**	**
Gervaise[14]	Retrospective cohort study	ER, with CTPA	**	*	**
Helms[15]	Prospective cohort study	ICU	****	**	**
Hippensteel[16]	Retrospective cohort study	ICU	****	**	**
Klok[17]	Prospective cohort study	ICU	****	**	**
Koleilat[18]	Retrospective case control study	Hospitalized	****	**	**
Larsen[19]	Prospective cohort study	Hospitalized returned travelers	**	*	**
Llitjos[20]	Retrospective cohort study	ICU	****	**	**
Lodigiani[21]	Retrospective cohort study	Hospitalized	****	**	**
Longchamp[22]	Retrospective cohort study	ICU	****	**	**
Mestre-Gomez[23]	Retrospective cohort study	Non-ICU	****	**	**
Monfardini[24]	Case series	Hospitalized	****	**	*
Nahum[25]	Case series	ICU	***	*	**
Patell[26]	Retrospective cohort study	Hospitalized	****	**	**
Poissy[27]	Cross sectional	ICU	****	**	**
Poyiadji[28]	Retrospective cohort study	Hospitalized, with CTPA	***	*	**
Rieder[29]	Prospective Cohort study	Hospitalized	****	**	**
Ren[30]	Cross-sectional study	ICU	***	**	*
Santoliquido[31]	Prospective Cohort study	Non-ICU	***	**	**
Soumagne[32]	Post hoc analysis of COVADIS	ICU	****	**	**

(*Continued*)

Supplementary Table S2 (*Continued*)

Author	Study design	Health care settings	Selection	Compatibility	Outcome
Stoneham[33]	Retrospective cohort study	Non-ICU	****	**	**
Trigonis[34]	Retrospective cohort study	ICU, with U/S	***	*	**
Trimaille[35]	Retrospective cohort study	Non-ICU	****	**	**
Voicu[36]	Prospective cohort study	ICU	****	**	**
Whyte[37]	Retrospective cohort study	Hospitalized	****	**	**
Zerwes[38]	Prospective cohort study	ICU	****	**	**
Li Zhang[39]	Cross sectional study	Non-ICU, with U/S	***	*	**
Maatman[40]	Case series	ICU	****	**	**
Thomas[41]	Retrospective cohort study	ICU	****	**	**
Grillet[42]	Retrospective cohort study	Hospitalized	****	**	**
Alharthy[43]	Prospective cohort study	ICU	****	*	**
Hanif[44]	Retrospective cohort study	Hospitalized	****	**	**
Le Jeune[45]	Retrospective cohort study	Non-ICU	****	*	**
Leonard-lorant[46]	Retrospective cohort study	Hospitalized, with CTPA	***	*	**
Aleva[47]	Case series	ICU	****	**	**
Ierardi[48]	Prospective cohort study	Hospitalized	****	*	**
Bavaro[49]	Case series	Hospitalized, with CTPA	***	*	*
Criel[50]	Prospective cohort study	Hospitalized	***	**	*
Mattioli[51]	Retrospective cohort study	Non-ICU	****	**	**
Middledrop[52]	Retrospective cohort study	Hospitalized	****	**	**
Piagnerelli[53]	Prospective cohort study	ICU	***	**	**
Stessel[54]	Before and after study	ICU	****	**	**
Taccone[55]	Retrospective analysis of prospective data	ICU	***	**	**
Tavazzi[56]	Prospective cohort study	ICU	***	**	**
Bi[57]	Prospective cohort study	Hospitalized	****	**	**
Cattaneo[58]	Prospective cohort study	Non-ICU	****	**	**
Galeano-Valle[59]	Prospective cohort study	Non-ICU	****	**	**
Grandmaison[60]	Cross sectional study	Hospitalized	***	**	*
Giorgi-Pierfranceschi[61]	Cross sectional study	Non-ICU with U/S	***	**	*
Contou[62]	Retrospective cohort study	ICU	****	**	**
Alonso-Fernández[63]	Prospective cohort study	Hospitalized, with CTPA	**	*	**
Benito[64]	Prospective cohort study	Hospitalized	****	**	**

 © 2021. The Author(s).

Supplementary Table S2 (*Continued*)

Author	Study design	Health care settings	Selection	Compatibility	Outcome
Atalla[65]	Retrospective cohort study	ICU, U/S if D-Dimer high	***	**	**
Avruscio[66]	Prospective cohort study	Hospitalized	****	**	**
CHOI[67]	Retrospective cohort study	Hospitalized	****	**	**
Hill[68]	Retrospective cohort study	Hospitalized	****	**	**
Jimenez-Guiu[69]	Prospective cohort study	Non-ICU	****	**	**
Lemos[70]	Randomized controlled trail	ICU	Randomization 2	Blinding 0	Patients 1
Longhitano[71]	Retrospective cohort study	ICU	****	**	**
Longhitano[72]	Prospective cohort study	Non-ICU	****	*	**
Melazzini[73]	Retrospective cohort study	Hospitalized	****	**	**
Mouhat[74]	Retrospective cohort study	Hospitalized	***	*	**
Nadeem[75]	Retrospective cohort study	ICU	****	**	**
Pancani[76]	Prospective cohort study	Non-ICU	***	*	**
Pavoni[77]	Retrospective cohort study	ICU	****	**	**
Piazza[78]	Retrospective cohort study	Hospitalized	****	**	**
Pizzi[79]	Retrospective cohort study	Non-ICU	***	**	**
Prandoni[80]	Retrospective cohort study	Non-ICU	***	**	*
Soumagne 2[81]	Retrospective cohort study	ICU, with CTPA	***	*	**
Torres-Machorro[82]	Prospective cohort study	ICU	***	*	**
Al-samkari 2[83]	Retrospective cohort study	ICU	****	**	**
berg[84]	Retrospective cohort study	Hospitalized	****	**	**
Marquez[85]	Prospective cohort study	Hospitalized	****	**	**
Tan[86]	Retrospective cohort study	Hospitalized	****	**	**
Warrior[87]	Prospective cohort study	Hospitalized	****	**	**
Theprungsirikul[88]	Retrospective cohort study	Hospitalized	****	**	**
Camille[89]	Retrospective cohort study	ICU	****	**	**
Kodama[90]	Retrospective cohort study	Hospitalized	****	**	**
Jiménez-Soto[91]	Cross sectional study	Hospitalized	****	**	**

 © 2021. The Author(s).

References

1 Al-Samkari H, Karp Leaf RS, Dzik WH, et al. COVID-19 and coagulation: bleeding and thrombotic manifestations of SARS-CoV-2 infection. Blood 2020;136(04):489–500
2 Artifoni M, Danic G, Gautier G, et al. Systematic assessment of venous thromboembolism in COVID-19 patients receiving thromboprophylaxis: incidence and role of D-dimer as predictive factors. J Thromb Thrombolysis 2020;50(01):211–216
3 Beun R, Kusadasi N, Sikma M, Westerink J, Huisman A. Thromboembolic events and apparent heparin resistance in patients infected with SARS-CoV-2. Int J Lab Hematol 2020;42 (Suppl 1):19–20
4 Bilaloglu S, Aphinyanaphongs Y, Jones S, Iturrate E, Hochman J, Berger JS. Thrombosis in Hospitalized Patients With COVID-19 in a New York City Health System. JAMA 2020;324(08): 799–801
5 Bompard F, Monnier H, Saab I, et al. Pulmonary embolism in patients with COVID-19 pneumonia. Eur Respir J 2020;56(01): 17–20
6 Chen S, Zhang D, Zheng T, Yu Y, Jiang J. DVT incidence and risk factors in critically ill patients with COVID-19. J Thromb Thrombolysis 2020;•••:1–7
7 Cui S, Chen S, Li X, Liu S, Wang F. Prevalence of venous thromboembolism in patients with severe novel coronavirus pneumonia. J Thromb Haemost 2020;18(06):1421–1424
8 Demelo-Rodríguez P, Cervilla-Muñoz E, Ordieres-Ortega L, et al. Incidence of asymptomatic deep vein thrombosis in patients with COVID-19 pneumonia and elevated D-dimer levels. Thromb Res 2020;192(January):23–26
9 Desborough MJR, Doyle AJ, Griffiths A, Retter A, Breen KA, Hunt BJ. Image-proven thromboembolism in patients with severe COVID-19 in a tertiary critical care unit in the United Kingdom. Thromb Res 2020;193:1–4
10 Dubois-Silva Á, Barbagelata-López C, Mena Á, Piñeiro-Parga P, Llinares-García D, Freire-Castro S. Pulmonary embolism and screening for concomitant proximal deep vein thrombosis in noncritically ill hospitalized patients with coronavirus disease 2019. Intern Emerg Med 2020;15(05):865–870
11 Fauvel C, Weizman O, Trimaille A, et al; Critical Covid-19 France Investigators. Pulmonary embolism in COVID-19 patients: a French multicentre cohort study. Eur Heart J 2020;41(32): 3058–3068
12 Freund Y, Drogrey M, Miró Ò, et al. Association between Pulmonary Embolism and COVID-19 in ED patients Undergoing CTPA: the PEPCOV international retrospective study. Acad Emerg Med 2020;27:811–820
13 Fraissé M, Logre E, Pajot O, Mentec H, Plantefève G, Contou D. Thrombotic and hemorrhagic events in critically ill COVID-19 patients: a French monocenter retrospective study. Crit Care 2020;24(01):275
14 Gervaise A, Bouzad C, Peroux E, Helissey C. Acute pulmonary embolism in non-hospitalized COVID-19 patients referred to CTPA by emergency department. Eur Radiol 2020;30(11): 6170–6177
15 Helms J, Tacquard C, Severac F, et al; CRICS TRIGGERSEP Group (Clinical Research in Intensive Care and Sepsis Trial Group for Global Evaluation and Research in Sepsis) High risk of thrombosis in patients with severe SARS-CoV-2 infection: a multicenter prospective cohort study. Intensive Care Med 2020;46(06): 1089–1098
16 Hippensteel JA, Burnham EL, Jolley SE. Prevalence of venous thromboembolism in critically ill patients with COVID-19. Br J Haematol 2020;190(03):e134–e137
17 Klok FA, Kruip MJHA, van der Meer NJM, et al. Confirmation of the high cumulative incidence of thrombotic complications in critically ill ICU patients with COVID-19: An updated analysis. Thromb Res 2020;191:148–150
18 Koleilat I, Galen B, Choinski K, et al. Clinical characteristics of acute lower extremity deep venous thrombosis diagnosed by duplex in patients hospitalized for coronavirus disease 2019. J Vasc Surg Venous Lymphat Disord 2021;9(01):36–46
19 Larsen K, Coolen-Allou N, Masse L, et al. Detection of Pulmonary Embolism in Returning Travelers with Hypoxemic Pneumonia due to COVID-19 in Reunion Island. Am J Trop Med Hyg 2020;103 (02):844–846
20 Llitjos JF, Leclerc M, Chochois C, et al. High incidence of venous thromboembolic events in anticoagulated severe COVID-19 patients. J Thromb Haemost 2020;18(07):1743–1746
21 Lodigiani C, Iapichino G, Carenzo L, et al; Humanitas COVID-19 Task Force. Venous and arterial thromboembolic complications in COVID-19 patients admitted to an academic hospital in Milan, Italy. Thromb Res 2020;191:9–14
22 Longchamp A, Longchamp J, Manzocchi-Besson S, et al. Venous thromboembolism in critically Ill patients with COVID-19: Results of a screening study for deep vein thrombosis. Res Pract Thromb Haemost 2020;4(05):842–847
23 Mestre-Gómez B, Lorente-Ramos RM, Rogado J, et al. Incidence of pulmonary embolism in non-critically ill COVID-19 patients. Predicting factors for a challenging diagnosis. J Thromb Thrombolysis 2020;•••:2–8
24 Monfardini L, Morassi M, Botti P, et al. Pulmonary thromboembolism in hospitalised COVID-19 patients at moderate to high risk by Wells score: a report from Lombardy, Italy. Br J Radiol 2020;93 (1113):20200407
25 Nahum J, Morichau-Beauchant T, Daviaud F, et al. Venous Thrombosis Among Critically Ill Patients With Coronavirus Disease 2019 (COVID-19). JAMA Netw Open 2020;3(05):e2010478
26 Patell R, Bogue T, Bindal P, et al. Incidence of thrombosis and hemorrhage in hospitalized cancer patients with COVID-19. J Thromb Haemost 2020;18(09):2349–2357
27 Poissy J, Goutay J, Caplan M, et al; Lille ICU Haemostasis COVID-19 Group. Pulmonary Embolism in Patients With COVID-19: Awareness of an Increased Prevalence. Circulation 2020;142(02): 184–186
28 Poyiadji N, Cormier P, Patel PY, et al. Acute Pulmonary Embolism and COVID-19. Radiology 2020;297(03):E335–E338
29 Rieder M, Goller I, Jeserich M, et al. Rate of venous thromboembolism in a prospective all-comers cohort with COVID-19. J Thromb Thrombolysis 2020;50(03):558–566
30 Ren B, Yan F, Deng Z, et al. Extremely High Incidence of Lower Extremity Deep Venous Thrombosis in 48 Patients With Severe COVID-19 in Wuhan. Circulation 2020;142(02):181–183
31 Santoliquido A, Porfidia A, Nesci A, et al; GEMELLI AGAINST COVID-19 Group. Incidence of deep vein thrombosis among non-ICU patients hospitalized for COVID-19 despite pharmacological thromboprophylaxis. J Thromb Haemost 2020;18(09): 2358–2363
32 Soumagne T, Lascarrou JB, Hraiech S, et al. Factors Associated With Pulmonary Embolism Among Coronavirus Disease 2019 Acute Respiratory Distress Syndrome: A Multicenter Study Among 375 Patients. Crit Care Explor 2020;2(07):e0166
33 Stoneham SM, Milne KM, Nuttall E, et al. Thrombotic risk in COVID-19: a case series and case-control study. Clin Med (Lond) 2020;20(04):e76–e81
34 Trigonis RA, Holt DB, Yuan R, et al. Incidence of Venous Thromboembolism in Critically Ill Coronavirus Disease 2019 Patients Receiving Prophylactic Anticoagulation. Crit Care Med 2020;48 (09):e805–e808
35 Trimaille A, Curtiaud A, Marchandot B, et al. Venous thromboembolism in non-critically ill patients with COVID-19 infection. Thromb Res 2020;193:166–169
36 Voicu S, Bonnin P, Stépanian A, et al. High Prevalence of Deep Vein Thrombosis in Mechanically Ventilated COVID-19 Patients. J Am Coll Cardiol 2020;76(04):480–482

 © 2021. The Author(s).

37 Whyte MB, Kelly PA, Gonzalez E, Arya R, Roberts LN. Pulmonary embolism in hospitalised patients with COVID-19. Thromb Res 2020;195:95–99

38 Zerwes S, Hernandez Cancino F, Liebetrau D, et al. [Increased risk of deep vein thrombosis in intensive care unit patients with CoViD-19 infections?-Preliminary data] Chirurg 2020;91(07): 588–594

39 Zhang L, Feng X, Zhang D, et al. Deep Vein Thrombosis in Hospitalized Patients With COVID-19 in Wuhan, China: Prevalence, Risk Factors, and Outcome. Circulation 2020;142(02): 114–128

40 Maatman TK, Jalali F, Feizpour C, et al. Routine Venous Thromboembolism Prophylaxis May Be Inadequate in the Hypercoagulable State of Severe Coronavirus Disease 2019. Crit Care Med 2020;48 (09):e783–e790

41 Thomas W, Varley J, Johnston A, et al. Thrombotic complications of patients admitted to intensive care with COVID-19 at a teaching hospital in the United Kingdom. Thromb Res 2020;191:76–77

42 Grillet F, Behr J, Calame P, Aubry S, Delabrousse E. Acute Pulmonary Embolism Associated with COVID-19 Pneumonia Detected with Pulmonary CT Angiography. Radiology 2020;296(03): E186–E188

43 Alharthy A, Faqihi F, Abuhamdah M, et al. Prospective Longitudinal Evaluation of Point-of-Care Lung Ultrasound in Critically Ill Patients With Severe COVID-19 Pneumonia. J Ultrasound Med 2020;2019:1–14

44 Hanif A, Khan S, Mantri N, et al. Thrombotic complications and anticoagulation in COVID-19 pneumonia: a New York City hospital experience. Ann Hematol 2020;99(10):2323–2328

45 Le Jeune S, Suhl J, Benainous R, et al. High prevalence of early asymptomatic venous thromboembolism in anticoagulated COVID-19 patients hospitalized in general wards. J Thromb Thrombolysis 2020

46 Léonard-Lorant I, Delabranche X, Séverac F, et al. Acute Pulmonary Embolism in Patients with COVID-19 at CT Angiography and Relationship to d-Dimer Levels. Radiology 2020;296(03): E189–E191

47 Aleva FE, van Mourik L, Broeders MEAC, Paling AJ, de Jager CPC. COVID-19 in critically ill patients in North Brabant, the Netherlands: Patient characteristics and outcomes. J Crit Care 2020;60(January):111–115

48 Ierardi AM, Coppola A, Fusco S, et al. Early detection of deep vein thrombosis in patients with coronavirus disease 2019: who to screen and who not to with Doppler ultrasound? J Ultrasound 2020:1–9

49 Bavaro DF, Poliseno M, Scardapane A, et al. Occurrence of Acute Pulmonary Embolism in COVID-19-A case series. Int J Infect Dis 2020;98(January):225–226

50 Criel M, Falter M, Jaeken J, et al. Venous thromboembolism in SARS-CoV-2 patients: only a problem in ventilated ICU patients, or is there more to it? Eur Respir J 2020;56(01):8–11

51 Mattioli M, Benfaremo D, Mancini M, et al. Safety of intermediate dose of low molecular weight heparin in COVID-19 patients. J Thromb Thrombolysis 2020;19(0123456789)

52 Middeldorp S, Coppens M, van Haaps TF, et al. Incidence of venous thromboembolism in hospitalized patients with COVID-19. J Thromb Haemost 2020;18(08):1995–2002

53 Piagnerelli M, Cauchie P, Vancutsem M, et al. Thromboprophylaxis in Critically Ill Coronavirus Disease 2019 Patients. Crit Care Explor 2020;2(08):e0177

54 Stessel B, Vanvuchelen C, Bruckers L, et al. Impact of implementation of an individualised thromboprophylaxis protocol in critically ill ICU patients with COVID-19: A longitudinal controlled before-after study. Thromb Res 2020;194:209–215

55 Taccone FS, Gevenois PA, Peluso L, et al. Higher Intensity Thromboprophylaxis Regimens and Pulmonary Embolism in Critically Ill Coronavirus Disease 2019 Patients. Crit Care Med 2020;48(11): e1087–e1090

56 Tavazzi G, Civardi L, Caneva L, Mongodi S, Mojoli F. Thrombotic events in SARS-CoV-2 patients: an urgent call for ultrasound screening. Intensive Care Med 2020;46(06):1121–1123

57 Bi Q, Hong C, Meng J, et al. Characterizing clinical progression of COVID-19 among patients in Shenzhen, China: an observational cohort study. medRxiv 2020:2020.04.22.20076190-2020.04.22

58 Marco C, Elena M, Bertinato S, et al. Pulmonary Embolism or Pulmonary Thrombosis in COVID-19? Is the Recommendation to Use High- Dose Heparin for Thromboprophylaxis Justified. Thromb Res 2020;120(08):1230–1232

59 Galeano-Valle F, Oblitas CM, Ferreiro-Mazón MM, et al. Antiphospholipid antibodies are not elevated in patients with severe COVID-19 pneumonia and venous thromboembolism. Thromb Res 2020;192(January):113–115

60 Grandmaison G, Andrey A, Périard D, et al. Systematic Screening for Venous Thromboembolic Events in COVID-19 Pneumonia. TH Open 2020;4(02):e113–e115

61 Giorgi-Pierfranceschi M, Paoletti O, Pan A, et al. Prevalence of asymptomatic deep vein thrombosis in patients hospitalized with SARS-CoV-2 pneumonia: a cross-sectional study. Intern Emerg Med 2020;15(08):1425–1433

62 Contou D, Pajot O, Cally R, et al. Pulmonary embolism or thrombosis in ARDS COVID-19 patients: A French monocenter retrospective study. PLoS One 2020;15(08):e0238413

63 Alonso-Fernández A, Toledo-Pons N, Cosío BG, et al. Prevalence of pulmonary embolism in patients with COVID-19 pneumonia and high D-dimer values: A prospective study. PLoS One 2020;15(08): e0238216

64 Benito N, Filella D, Mateo J, et al. Pulmonary Thrombosis or Embolism in a Large Cohort of Hospitalized Patients With Covid-19. Front Med (Lausanne) 2020;7(August):557

65 Atallah B, Sadik ZG, Salem N, et al. The impact of protocol-based high-intensity pharmacological thromboprophylaxis on thrombotic events in critically ill COVID-19 patients. Anaesthesia 2020

66 Avruscio G, Camporese G, Campello E, et al; COVID-VTE Study Group. COVID-19 and Venous Thromboembolism in Intensive Care or Medical Ward. Clin Transl Sci 2020;13(06):1108–1114

67 Choi JJ, Wehmeyer GT, Li HA, et al. D-dimer cut-off points and risk of venous thromboembolism in adult hospitalized patients with COVID-19. Thromb Res 2020;196:318–321

68 Hill JB, Garcia D, Crowther M, et al. Frequency of venous thromboembolism in 6513 patients with COVID-19: a retrospective study. Blood Adv 2020;4(21):5373–5377

69 Jimenez-Guiu X, Huici-Sánchez M, Romera-Villegas A, Izquierdo-Miranda A, Sancho-Cerro A, Vila-Coll R. Deep vein thrombosis in non-critically ill patients with coronavirus disease 2019 pneumonia: deep vein thrombosis in non-intensive care unit patients. J Vasc Surg Venous Lymphat Disord 2020:S2213-333X(20)30466-2

70 Lemos ACB, do Espírito Santo DA, Salvetti MC, et al. Therapeutic versus prophylactic anticoagulation for severe COVID-19: A randomized phase II clinical trial (HESACOVID). Thromb Res 2020; 196:359–366

71 Longhitano Y, Racca F, Zanza C, et al. Venous thromboembolism in critically ill patients affected by ARDS related to COVID-19 in Northern-West Italy. Eur Rev Med Pharmacol Sci 2020;24(17): 9154–9160

72 Longhitano Y, Racca F, Zanza C, et al. Venous Thrombo-Embolism in Hospitalized SARS-CoV-2 Patients Treated with Three Different Anticoagulation Protocols: Prospective Observational Study. Biology (Basel) 2020;9(10):E310

73 Melazzini F, Colaneri M, Fumoso F, et al; San Matteo Pavia COVID-19 Task Force. Venous thromboembolism and COVID-19: a single center experience from an academic tertiary referral hospital of Northern Italy. Intern Emerg Med 2020

74 Mouhat B, Besutti M, Bouiller K, et al. Elevated D-dimers and lack of anticoagulation predict PE in severe COVID-19 patients. Eur Respir J 2020;56(04):2001811

 © 2021. The Author(s).

75 Nadeem R, Thomas SJ, Fathima Z, et al. Pattern of anticoagulation prescription for patients with Covid-19 acute respiratory distress syndrome admitted to ICU. Does it impact outcome? Heart Lung 2021;50(01):1–5
76 Pancani R, Villari L, Foci V, et al. Lower limb deep vein thrombosis in COVID-19 patients admitted to intermediate care respiratory units. Thromb Res 2021;197(197):44–47
77 Pavoni V, Gianesello L, Pazzi M, Stera C, Meconi T, Frigieri FC. Venous thromboembolism and bleeding in critically ill COVID-19 patients treated with higher than standard low molecular weight heparin doses and aspirin: A call to action. Thromb Res 2020; 196:313–317
78 Piazza G, Campia U, Hurwitz S, et al. Registry of Arterial and Venous Thromboembolic Complications in Patients With COVID-19. J Am Coll Cardiol 2020;76(18):2060–2072
79 Pizzi R, Gini G, Caiano L, et al. Coagulation parameters and venous thromboembolism in patients with and without COVID-19 admitted to the Emergency Department for acute respiratory insufficiency. Thromb Res 2020;196(December):209–212
80 Prandoni P, Cattelan AM, Carrozzi L, et al; FONDACOVIT Investigators [all in Italy]. The hazard of fondaparinux in non-critically ill patients with COVID-19: Retrospective controlled study versus enoxaparin. Thromb Res 2020;196:395–397
81 Soumagne T, Winiszewski H, Besch G, et al. Pulmonary embolism among critically ill patients with ARDS due to COVID-19. Respir Med Res 2020;78:100789
82 Torres-Machorro A, Anguiano-Álvarez VM, Grimaldo-Gómez FA, et al. Asymptomatic deep vein thrombosis in critically ill COVID-19 patients despite therapeutic levels of anti-Xa activity. Thromb Res 2020;196:268–271
83 Al-Samkari HGS, Karp Leaf R, Wang W, Rosovsky R, Bauer K, Leaf D. STOP-COVID Investigators. Thrombosis, Bleeding, and the Effect of Anticoagulation on Survival in Critically Ill Patients with COVID-19 in the United States. . [abstract]Res Pract Thromb Haemost 2020;4(Suppl 1
84 Berg S, Kim S-H, Patel S, et al. Real World Outcomes of Sars-Cov-2 Thrombosis Rates across Three University Health Systems in the Chicago Metropolitan Area. Blood 2020;136(Suppl 1):58–59
85 Marquez FG, Riviello-Goya S, Vargas Ruiz AG, et al. Low Rate of Thrombosis in Mexican Patients with COVID-19 Infection. a Benefit of Higher Doses Anticoagulants or a Sub Diagnosis? Blood 2020;136(Suppl 1):29–30
86 Tan JY, Tan CW, Wong WH, et al. Thrombotic Events in COVID-19 Patients and Its Comparison with Other Non-Sars-Cov-2 Respiratory Viruses. Blood 2020;136(Suppl 1):25–26
87 Warrior S, Behrens E, Thomas J, et al. Impact of Treatment and Anticoagulation on Thrombosis in COVID-19 Patients. Blood 2020;136(Suppl 1):6–7
88 Theprungsirikul PP, Saith SE. Incidence of Thrombosis and Associated Risk Factors in Hospitalized COVID-19 Patients in a New York City Hospital System. Blood 2020;136(Suppl 1):32–33
89 Camille S, Koolian M, Escobar D, et al. Thrombotic Complications in a Canadian Population of Critically Ill Patients with COVID-19. Blood 2020;136(Suppl 1):41–42
90 Kodama R, Kalsi A, Singh A, et al. Outcomes in COVID-19 Patients on Treatment Dose Anti-Coagulation Compared to Prophylactic Dose Anti-Coagulation. Blood 2020;136(Suppl 1):40–41
91 Jiménez-Soto R, Aguilar-Soto M, Demichelis R. The Impact of Different Prophylactic Anticoagulation Doses on the Outcomes of Patients with COVID-19. Blood 2020;136(Suppl 1):17

 © 2021. The Author(s).
